# Massive Bilateral Pulmonary Thromboembolism Due to a Thrombus in Transit to the Right Atrium: A Case Report

**DOI:** 10.7759/cureus.101643

**Published:** 2026-01-15

**Authors:** Sharon Hefziba Pineda Guevara, César Alas-Pineda, Carlos Alvarado, José Arturo Portillo, Danny Istayul Rivera Rodriguez, Sendy Ruiz

**Affiliations:** 1 Department of Internal Medicine, Dr. Mario Catarino Rivas National Hospital, San Pedro Sula, HND; 2 University School of Health Sciences, National Autonomous University of Honduras, San Pedro Sula, HND; 3 Department of Analytics, Ferrer Pulmonary Institute, Hallandale Beach, FL, USA; 4 Department of Quantitative Biomedical Sciences, Dartmouth College, Hanover, USA; 5 Faculty of Medicine and Surgery, Catholic University of Honduras, San Pedro Sula, HND; 6 Department of Internal Medicine, Honduran Social Security Institute, San Pedro Sula, HND

**Keywords:** echocardiography, pulmonary embolism (pe), thrombus in transit

## Abstract

A 48-year-old man presented to a rural Honduran hospital with progressive dyspnea for three days, worsening at rest. He was hemodynamically stable and without tachypnea or pulmonary crackles. The electrocardiogram showed sinus tachycardia, suggestive of pulmonary thromboembolism (PTE). Chest CT confirmed the diagnosis of bilateral PTE with total occlusion of the right pulmonary artery and significant stenosis of the left pulmonary artery. Transthoracic echocardiogram revealed a right atrial thrombus. Medical treatment with therapeutic anticoagulation was provided. Echocardiographic follow-up at 10 days showed that the original thrombus had been significantly reduced to two smaller thrombi. The patient was discharged from the hospital with more anticoagulants. Thrombus in transit alongside pulmonary embolism is rare and is an emergent condition; it can quickly progress to cardiogenic shock or cardiac arrest. This patient’s effective diagnosis and treatment highlight the importance of bedside echocardiography in resource-limited settings.

## Introduction

Right atrial (RA) thrombus associated with pulmonary embolism (PE) is an uncommon clinical entity, with an estimated annual incidence of approximately 1 per 1,000 individuals. Deep venous thrombosis (DVT) and PE frequently coexist; approximately 33% to 73% of patients with DVT have concurrent PE, while 51.2% to 97% of patients diagnosed with PE present with concomitant DVT [1]. Clinical presentation varies widely, ranging from PE with mild respiratory symptoms to massive PE, defined by the presence of systemic hypotension or cardiogenic shock [2].

A thrombus in transit is defined as a thrombus temporarily lodged within the cardiac chambers. Given the broad spectrum of clinical manifestations, the diagnosis of thrombus in transit and PE is challenging and requires a high index of suspicion, along with the integration of multiple diagnostic modalities, including transthoracic and transesophageal echocardiography, computed tomography (CT) angiography, and cardiac magnetic resonance imaging (MRI) [2,3].

Management of this complex condition is equally challenging. Thrombolysis and thrombectomy are associated with the highest survival rates, with reported success rates of up to 80% and 90%, respectively [4,5]. However, the final therapeutic decision must carefully consider the individual patient context, including bleeding risk, the likelihood of recurrent thromboembolism, and the presence of comorbidities [6].

## Case presentation

**Figure 1:**
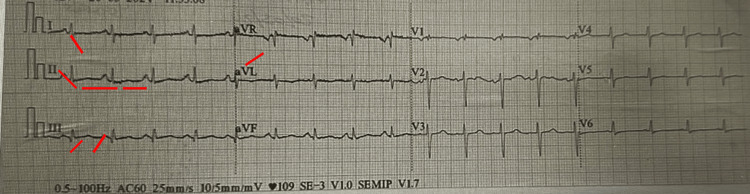
Twelve-lead echocardiogram. Sinus rhythm; heart rate (HR) 100 bpm; +60°; PR interval 0.16 s; QRS duration 0.08 s; S1Q3T3 pattern with incomplete right bundle branch block.

A 48-year-old man presented to the emergency department with progressive dyspnea for three days, worsening at rest. His blood pressure was 120/90 mmHg, heart rate 123 beats per minute, respiratory rate 24 breaths per minute, and oxygen saturation was normal on room air. He had no signs of hemodynamic instability and no history of heart disease. The electrocardiogram showed sinus tachycardia and an S1Q3T3 pattern compatible with the McGinn-White sign (Figure 1).

**Figure 2:**
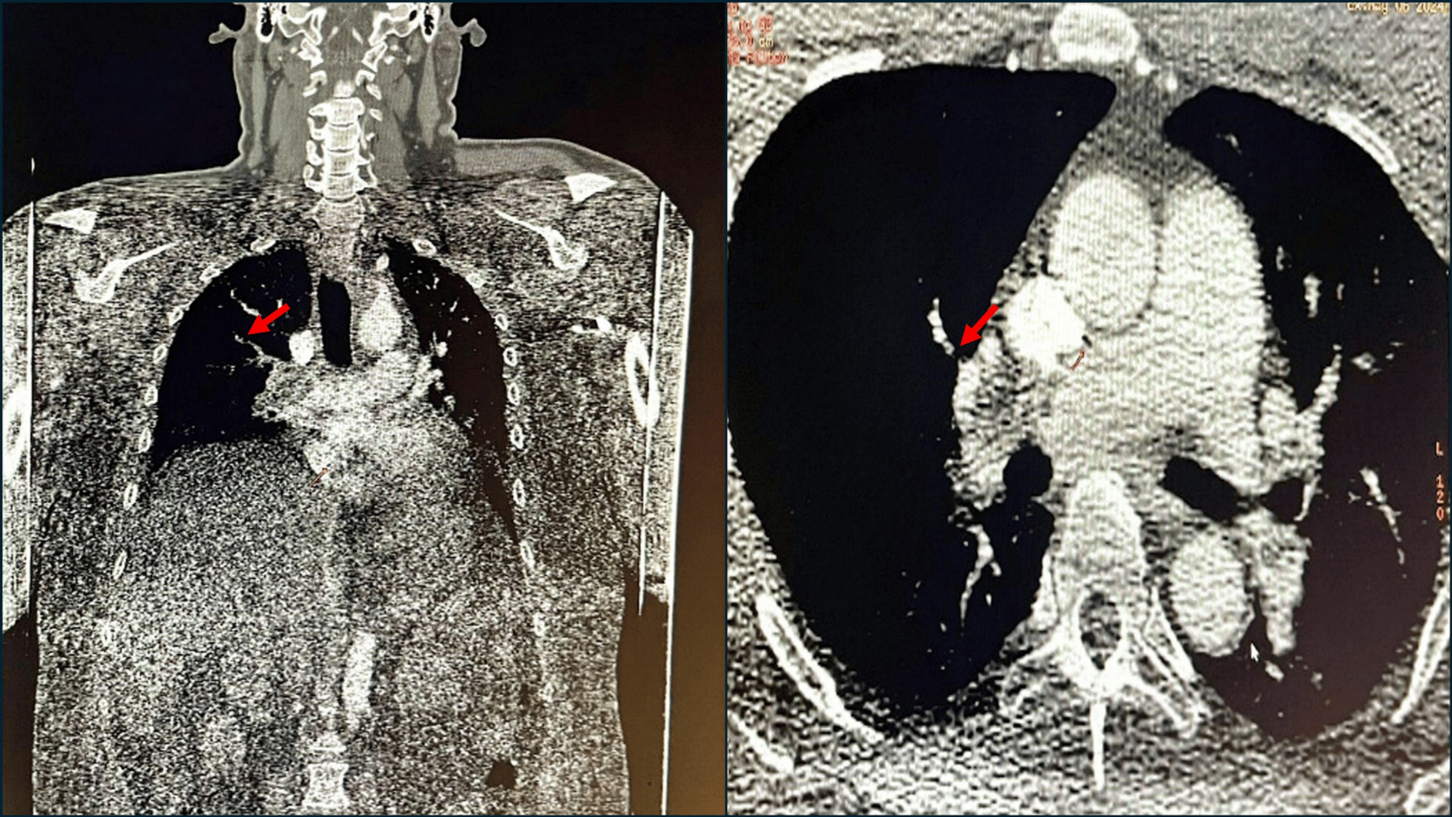
Thoracic CT angiography. (A) Coronal and (B) axial sections demonstrating bilateral pulmonary thromboembolism, with obstruction of both main pulmonary arteries (red arrows).

Pulmonary CT angiography revealed massive bilateral pulmonary thrombosis with total occlusion of the right pulmonary artery and significant stenosis of the left pulmonary artery (Figures 2A-2B). Complete obstruction of the right pulmonary branch and the middle and inferior segmental branches was also observed.

**Figure 3:**
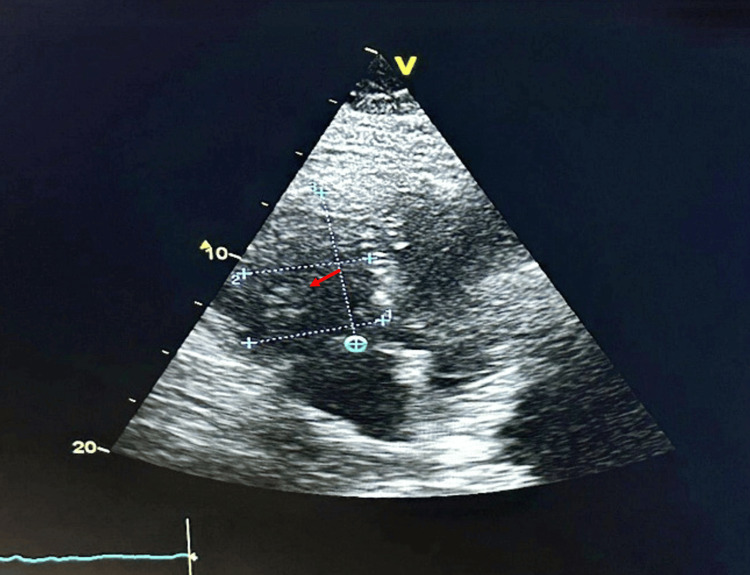
Echocardiogram showing a thrombus in the right atrium, visible as a mobile mass within the atrial cavity (red arrow).

Echocardiography identified a 2 × 4 cm thrombus extending from the superior vena cava and protruding into the right ventricle during systole (Figure 3A). Right ventricular dilation was observed, with systolic function parameters including tricuspid annular plane systolic excursion (TAPSE) of 16 mm, tricuspid regurgitation (TR) velocity of 17 cm/s, and a shortening fraction of 25%. The Pulmonary Embolism Severity Index (PESI) score of 98 classified the patient as intermediate-high risk.

Based on these findings, the medical team discussed treatment options. The patient refused intravenous thrombolysis, and the cardiovascular team determined he was not a surgical candidate. Therefore, medical treatment with enoxaparin 1 mg/kg every 12 hours was initiated.

After 10 days of treatment, a follow-up echocardiogram (Video 1) showed a significant reduction in the size of the thrombus. Two residual thrombi remained: one at the superior vena cava-right atrium junction (0.8 × 1 cm) and one in the right ventricle near the subvalvular apparatus (0.9 × 0.7 cm). Follow-up CT demonstrated complete resolution of the massive pulmonary thrombosis and normalization of the RV/LV ratio. The patient was discharged without dyspnea or signs of heart failure, with outpatient treatment consisting of apixaban 5 mg every 12 hours, atorvastatin 40 mg daily, amlodipine 10 mg daily, dapagliflozin 10 mg daily, and candesartan 16 mg daily.

**Video 1:** Apical approximation of the right ventricle. The follow-up study shows almost complete resolution of the thrombus, with no evidence of residual mobile intracavitary masses.

At the 30-day follow-up, the patient remained stable, with no complications or recurrence.

## Discussion

Right venous thrombi in transit are deep venous thrombi that have embolized and become temporarily lodged in the right-sided cardiac chambers while en route to the pulmonary arteries. These thrombi may further migrate, resulting in additional embolic complications. This condition is exceedingly rare and, if left untreated, carries a mortality rate ranging from 80% to 100% [7].

We report the case of a 48-year-old patient with pulmonary thromboembolism (PTE) involving the main pulmonary branches, accompanied by a thrombus in the right atrium extending into the right ventricle [2,8]. Management consisted of anticoagulation with enoxaparin as a secondary therapeutic strategy.

Pulmonary embolism itself is a severe condition with a mortality rate of 26%-30% when untreated; however, its concomitant presentation with a thrombus in transit is rare and potentially fatal, with mortality rates of 80%-100% [9]. In this case, timely treatment with anticoagulation, initially enoxaparin followed by apixaban, proved effective in resolving the thrombus and preventing further complications.

Although no standardized treatment algorithm exists, reviews comparing outcomes among therapeutic modalities demonstrate that mortality associated with anticoagulation alone is approximately 37%. In contrast, surgical thrombectomy and thrombolysis are associated with mortality rates of 18.3% and 13.7%, respectively [9]. Consequently, when feasible and with patient consent, thrombolysis or surgical thrombectomy is recommended as first-line therapy due to their superior effectiveness [2,10]. Nevertheless, anticoagulation remains a viable option in patients without access to or eligibility for other interventions. In our patient, a follow-up echocardiogram performed 10 days after initiating enoxaparin at a dose of 1 mg/kg demonstrated a significant reduction in thrombus size. This case underscores the critical importance of early diagnosis and timely therapeutic decision-making in patients with thrombus in transit and massive bilateral PTE.

It is important to highlight that ethnic background, the presence of concomitant risk factors, and residence in resource-limited settings, including Honduras, the patient’s country of origin, are associated with a high incidence and mortality from cardiovascular diseases (CVDs) [11]. One contributing factor is limited access to advanced cardiac diagnostic modalities. Multiple studies have shown that Hispanic populations not only exhibit high rates of hypertension, obesity, diabetes, and tobacco use, but also face significant socioeconomic barriers; lower socioeconomic status is associated with worse cardiovascular outcomes [11]. Compared with other Latin American countries, Honduras has a minimal number of cardiac imaging scanners, including CT, MRI, and positron emission tomography-CT (PET-CT), as well as a shortage of physicians trained in nuclear medicine, which significantly hinders accurate diagnosis and appropriate management of patients presenting with cardiac complications [5]. These disparities are partly attributable to the high cost of medical equipment and insufficient awareness of appropriate diagnostic strategies.

Given its low cost, widespread availability, even in resource-limited settings, and ease of use, point-of-care transthoracic echocardiography (TTE) is the preferred diagnostic modality for thrombus in transit, surpassing other imaging techniques such as CT angiography and cardiac MRI [5]. Thrombus in transit may present with sudden retrosternal chest pain and dyspnea, along with echocardiographic findings of right-sided chamber dilation, pulmonary hypertension, and a mobile intracardiac thrombus. TTE is particularly effective for detecting thrombi in transit because it enables visualization of both in situ thrombi and emboli actively traversing the heart [5,8].

Although the use of ultrasound is increasing across low- and middle-income countries, access remains limited in rural and underserved regions. Furthermore, there is a notable scarcity of published literature addressing the use of TTE and other echocardiographic modalities in Central American countries [12]. Expanded research on the implementation of TTE in Honduras and the broader Central American region may improve early diagnosis and clinical outcomes for patients presenting with complex and emergent cardiovascular conditions.

## Conclusions

This case represents an exceptional presentation of bilateral pulmonary embolism accompanied by a thrombus in transit. Despite the presence of an extensive thrombotic burden documented by angiography and echocardiography, there was no evidence of hemodynamic compromise or hypoxemia. Although infrequent, this condition is associated with high mortality and requires prompt diagnosis and treatment. Owing to contraindications to thrombolysis and surgical intervention, a conservative anticoagulation-based management approach was required, which alone proved effective in achieving thrombus resolution.

It is relevant to emphasize the importance of a comprehensive and individualized patient evaluation, providing clinical evidence that in carefully selected scenarios, anticoagulation may represent an effective therapeutic strategy even in anatomically massive presentations.

Bedside echocardiography played a pivotal role in both diagnosis and serial follow-up, underscoring its value in resource-limited settings.
